# A fast and highly sensitive blood culture PCR method for clinical detection of *Salmonella enterica *serovar Typhi

**DOI:** 10.1186/1476-0711-9-14

**Published:** 2010-04-19

**Authors:** Liqing Zhou, Andrew J Pollard

**Affiliations:** 1 Oxford Vaccine Centre, University of Oxford, Oxford, UK

## Abstract

**Background:**

*Salmonella *Typhi causes an estimated 21 million new cases of typhoid fever and 216,000 deaths every year. Blood culture is currently the gold standard for diagnosis of typhoid fever, but it is time-consuming and takes several days for isolation and identification of causative organisms. It is then too late to initiate proper antibiotic therapy. Serological tests have very low sensitivity and specificity, and no practical value in endemic areas. As early diagnosis of the disease and prompt treatment are essential for optimal management, especially in children, a rapid sensitive detection method for typhoid fever is urgently needed. Although PCR is sensitive and rapid, initial research indicated similar sensitivity to blood culture and lower specificity. We developed a fast and highly sensitive blood culture PCR method for detection of *Salmonella *Typhi, allowing same-day initiation of treatment after accurate diagnosis of typhoid.

**Methods:**

An ox bile tryptone soy broth was optimized for blood culture, which allows the complete lysis of blood cells to release intracellular bacteria without inhibiting the growth of *Salmonella *Typhi. Using the optimised broth *Salmonella *Typhi bacteria in artificial blood samples were enriched in blood culture and then detected by a PCR targeting the *fliC-d *gene of *Salmonella *Typhi.

**Results:**

Tests demonstrated that 2.4% ox bile in blood culture not only lyzes blood cells completely within 1.5 hours so that the intracellular bacteria could be released, but also has no inhibiting effect on the growth of *Salmonella *Typhi.

Three hour enrichment of *Salmonella *Typhi in tryptone soya broth containing 2.4% ox bile could increase the bacterial number from 0.75 CFU per millilitre of blood which is similar to clinical typhoid samples to the level which regular PCR can detect. The whole blood culture PCR assay takes less than 8 hours to complete rather than several days for conventional blood culture.

**Conclusions:**

This novel blood culture PCR method is superior in speed and sensitivity to both conventional blood culture and PCR assays. Its use in clinical diagnosis may allow early detection of the causative organism and facilitate initiation of prompt treatment among patients with typhoid fever.

## Introduction

*Salmonella enterica *serovar Typhi, the human-specific, causative agent of typhoid fever, causes an estimated 21 million new cases and 216,000 deaths every year [1]. It is generally thought that *Salmonella enterica *serovar Typhi, an enterically acquired invasive pathogen, penetrates the ileal epithelium and is transported via underlying macrophages to spleen, liver, and other target tissues during the normal disease course. Natural clearance of the disease is thought to entail development of specific humoral and cellular immune responses. However, the precise mechanisms of *Salmonella *serovar Typhi virulence and immune protection are not well understood, mainly due to the absence of a suitable animal model. We have been developing a human typhoid challenge model which could increase understanding of the pathogenesis of typhoid fever, the immunobiology of the disease and the correlates of protection, and provide a model for evaluation of new vaccines. In the case of *Salmonella *serovar Typhi, there is an urgent need for new improved vaccines, as those currently available all have limitations. In the planned clinical typhoid challenge study the volunteers will receive oral inoculation with wild-type *Salmonella *serovar Typhi, followed by monitoring for 14 days to detect the presence and load of *Salmonella *serovar Typhi in blood, which requires a robust, rapid and sensitive method of detection of *Salmonella *serovar Typhi.

Currently there is lack of reliable, rapid and sensitive methods for the clinical detection *Salmonella *serovar Typhi. In the regions where enteric fever is common, clinical diagnosis of typhoid fever is inadequate, as the symptoms it causes are non-specific and overlap with those of other febrile illness, such as vector-borne malaria, dengue fever and rickettsioses as well as environmentally transmitted leptospirosis and melioidosis [2,3]. Serological tests, predominantly the Widal test, are available but have very low sensitivity and specificity, and no practical value in endemic areas despite their continued use [4]. Isolation of the causative organism remains the most effective diagnostic method in suspected typhoid fever and blood has been the main sample for culture for *Salmonella *serovar Typhi since 1900 [5,6]. The sensitivity of blood culture is highest in the first week of the illness and reduces with advancing illness [7]. However, blood culture is time-consuming and takes at least 2 to 5 days until the identification of the organism. Blood culture can identify 45 to 70% of patients with typhoid fever, depending on the amount of blood sampled, the bacteraemic level of *Salmonella *serovar Typhi, the type of culture medium used, and the length of incubation period [8,9]. Several factors may contribute to failure to isolate the organism from blood, including inadequate laboratory media, intrinsic bactericidal activity of blood, the volume of blood taken for culture, the presence of antibiotics and the time of blood collection. The intracellular nature of *Salmonella *serovar Typhi also slows its growth in blood culture media. One study found that more than 50% of bacterial cells were present intracellularly in the blood from patients with typhoid fever [8]. In the first review of blood culture in typhoid fever in 1907 Coleman recommended the use of ox bile broth [10]. Its superior qualities were attributed to the inhibition of bactericidal activity of blood and its ability to release intracellular bacteria [11].

Given the problems associated with the diagnosis of typhoid fever by blood culture and serological methods, PCR methods were recently exploited. Several studies have been reported since the first evaluation of PCR as a diagnostic tool for typhoid fever in 1993 when Song et al. successfully amplified the flagellin gene (*fliC-d*) of *Salmonella *serovar Typhi in all cases of culture proven typhoid fever and from none of the healthy controls [12-18]. These studies reported excellent sensitivity and specificity when compared to positive (blood culture proven) and healthy controls. The clinical utility of these PCR tests was inadequately evaluated though some studies claimed that as few as 10 bacteria per millilitre of blood could be detected. The number of *Salmonella *bacteria circulating in the blood of a patient with *Salmonella *bacteremia is small. One study found 0.5-22 bacteria per millilitre of blood in 15 patients with typhoid fever [19], and another showed a median of 0.3 (IQR, 0.1-10) bacteria per millilitre of blood from 81 patients with typhoid fever [8]. The very low ratio of bacterial to human DNA means that the PCR template in clinical preparations is dominated by mammalian DNA and could cause false-positive PCR signals due to the non-specific binding of primers and false-negative results due to reduced sensitivity. In practice, the large excess of human DNA does indeed cause problems for PCR-based pathogen detection in blood, particularly in samples with low bacterial numbers [20,21]. Furthermore, small volumes of blood are often used for DNA extraction or as template in the PCR, which will significantly lower the sensitivity of these tests.

In the present study, we describe a fast and highly sensitive blood culture PCR method for detection of *Salmonella *serovar Typhi. The method uses the optimised ox bile-containing medium in blood culture for enrichment of bacteria, combined with a PCR assay, to reduce turnaround time for diagnosis and increase diagnostic sensitivity.

## Materials and methods

### Strains and culture

Wild-type *Salmonella *serovar Typhi Quailes strain was obtained from the University of Maryland through Centres for Disease Control and Prevention, Atlanta, USA. The strain was originally isolated from a carrier in 1958, and was previously used as a challenge strain over 20 years (in the 1960s and 1970s) during which almost 2000 volunteers ingested the strain as participants in vaccine efficacy and pathogenesis studies. The strain was last stored in 1988 and was maintained frozen until shipping to Oxford in 2008. In this study the strain was sub-cultured in tryptone soya broth (TSB) or on tryptone soya agar (TSA) (Oxoid, Basingstoke, UK) as needed.

### Preparation of *Salmonella *serovar Typhi Quailes strain inocula

*Salmonella *serovar Typhi Quailes strain was cultured on TSA plate for 16 hours at 37°C, and collected in TSB medium. The culture was first adjusted to an optical density at 600 nm of 0.25 and then prepared for serial 10-fold dilutions in TSB. The number of *Salmonella *bacteria per millilitre was determined by plating each dilution onto TSA plates. Colonies were counted after incubation of the plates overnight at 37°C.

### Growth of *Salmonella *serovar Typhi Quailes strain in TSB-bile medium

About 2-6 colony-forming-unit (CFU) of *Salmonella *serovar Typhi Quailes strain in 0.1 ml TSB was inoculated into 20 ml TSB each containing 0, 1.2, 1.6, 2.0, 2.4, 4.0 and 10.0% (w/v) of ox bile (Oxgall, BD Biosciences, Oxford, UK). The culture was shaken at 200 revolutions per minute (RPM) at 37°C in an incubator for 5 hours, and then centrifuged at 6,000 × g for 20 min. The pellet was re-suspended in 0.5 ml TSB and plated onto TSA plates. The plates were incubated overnight at 37°C and colonies were counted.

### Lysis of blood cells in TSB-bile medium

Blood was removed from healthy individuals with a sterile syringe and immediately pipetted into tubes containing heparin. 1 ml of blood was mixed with 4 ml of TSB each containing 0, 1.5, 2.0, 2.5, 3.0 and 5.0% (w/v) of ox bile, giving final concentrations 0, 1.2, 1.6, 2.0, 2.4 and 4.0% (w/v) of ox bile and 20% (v/v) of blood. The mixture was gently shaken for up to 5 hours at room temperature. 10 μl samples were taken periodically and mixed with 200 μl phosphate buffered saline (PBS). The lysis of blood cells was examined by microscopy.

### TSB-bile blood culture of *Salmonella *serovar Typhi Quailes strain

For blood culture of *Salmonella *serovar Typhi Quailes strain, 4 ml blood was spiked with 2-6 CFU of *Salmonella *serovar Typhi, and then mixed with 16 ml TSB containing 3.0% (w/v) ox bile in a 50 ml tube, giving a final concentration of ox bile at 2.4% (w/v) and blood at 20% (v/v). The culture was shaken at 200 RPM at 37°C in an incubator for up to 5 hours. Two tubes were removed from the incubator every hour and the cells were collected by centrifugation at 6,000 × g for 20 min. The supernatant was discarded and the pellet was re-suspended in 0.5 ml TSB. One tube was plated onto TSA plates for determination of the number of CFU in each sample and another tube was used for DNA extraction.

### DNA extraction

DNA was isolated from the culture using UltraClean™ BloodSpin™ Kit (MO BIO Laboratories, CA, USA) according to the manufacturer's instruction, except that the DNA was eluted with 100 μl Buffer 5 and incubated at 65°C for 5 min before centrifugation. The aliquots of DNA preparation were separated by electrophoresis on 1% agarose gels, stained with ethidium bromide, and photographed by a UV transilluminator. The quantity and quality of the DNA preparations were compared.

### PCR Primers of *Salmonella *serovar Typhi Quailes strain

The PCR primers for *Salmonella *serovar Typhi Quailes strain were designed according to the *fliC-d *gene sequence of *Salmonella *serovar Typhi (Accession number L21912): H-for (ACTCAGGCTTCCCGTAACGC) and Hd-rev (GGCTAGTATTGTCCTTATCGG) [22], and synthesized by Sigma Genosys (Sigma-Aldrich, Dorset, England).

### PCR protocol

The PCR reaction was carried out in a 50 μl volume, comprising 0.5 U of Taq DNA polymerase (Qiagen, Crawley, UK), 1 × Qiagen PCR buffer, 1.5 mM magnesium chloride, 200 μM concentrations of each deoxynucleoside triphosphate, 0.5 μM concentrations of the primers H-for and Hd-rev, and 10 μl of DNA template. The following amplification steps were used: 1 cycle of 95°C for 5 min; 35 cycles of 93°C for 30 sec, 55°C for 30 sec, and 72°C for 40 sec; and 1 cycle of 72°C for 5 min. The PCR amplification product was separated by electrophoresis on a 1% agarose gel, stained with ethidium bromide, and photographed by a UV transilluminator.

## Results

### Effect of ox bile on the growth of *Salmonella *serovar Typhi Quailes strain

**Figure 1:**
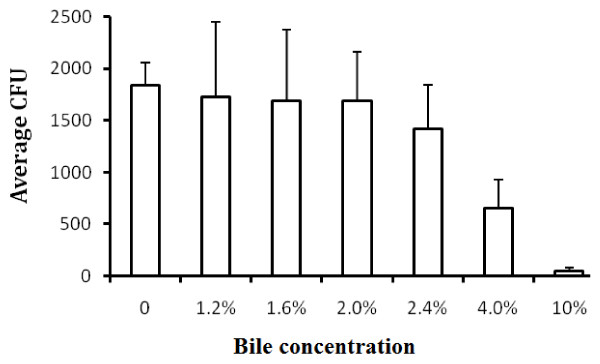
**The growth of *Salmonella *serovar Typhi Quailes strain in TSB containing different concentrations of ox bile**. Four bacteria were incubated in 20 ml TSB containing different concentrations of bile at 37°C for 5 hours. The CFU was the mean of three independent cultures.

The growth of *Salmonella *serovar Typhi Quailes strain in 20 ml TSB containing different concentrations of ox bile inoculated with four CFU is shown in Figure 1. There was no difference in the growth of *Salmonella *serovar Typhi Quailes strain in TSB with bile concentrations between 0 and 2.0%. At a bile concentration of 2.4%, the growth of *Salmonella *serovar Typhi Quailes strain became slower than those at the lower bile concentrations. However, 4.0% bile reduced the growth of *Salmonella *serovar Typhi Quailes strain by more than 50%, and eventually *Salmonella *serovar Typhi Quailes strain stopped growing when the bile concentration reached 10.0%.

### Effect of ox bile on blood cells

Bile rapidly and completely lyzed blood cells (see Table 1). No lysis of blood cells was found in TSB, and only partial lysis at a bile concentration of 1.2%, even after incubation for up to 5 hours. However, the blood cells were completely lyzed within 3 hours at a bile concentration of 1.6% and above. The higher the bile concentration, the faster the lysis of blood cells.

**Table 1:** Effect of ox bile on blood cells.

	Bile concentration (%)					
	
Incubation time (hour)	0	1.2	1.6	2.0	2.4	4.0
1						Complete

1.5					Complete	

2				Complete		

3			Complete			

5	No	Partial				

### Growth and PCR detection of *Salmonella *serovar Typhi Quailes strain in TSB-bile blood culture system

**Figure 2:**
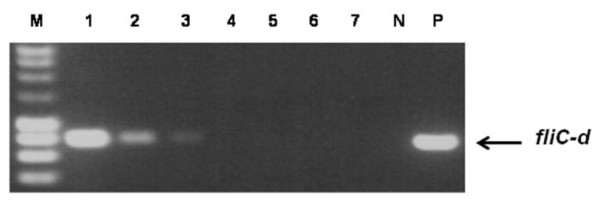
***Salmonella *serovar Typhi *fliC-d *amplicons (763 bp) on a 1% agarose gel**. Lanes: M, DNA marker; 1, 5 hour culture; 2, 4 hour culture; 3, 3 hour culture, 4, 2 hour culture; 5, 1 hour culture; 6, 0 hour culture; 7, No DNA template negative control; N, 5 hour blood culture control without bacteria; P, *Salmonella *serovar Typhi DNA positive control.

Based on the effect of bile on the growth of *Salmonella *serovar Typhi Quailes strain and the observation that bile did lyze blood cells, a TSB-bile blood culture system containing 2.4% ox bile and 20% blood was selected for *Salmonella *serovar Typhi Quailes strain blood culture. Table 2 shows the effect on CFU counts and PCR detection of *Salmonella *serovar Typhi Quailes strain using the TSB-bile blood culture system. The culture started from 3 CFU, and grew about 10 generations within 5 hours of incubation in this culture system. In the first hour the bacteria did not multiply, but the growth gradually picked up from the second hour. The number of CFU increased from 3 to 105 within 3 hours and rose more than thousand times during 5 hours of incubation. When the DNA, prepared periodically from the cultures, was used as PCR template, the amplicons of *fliC-d *gene of *Salmonella *serovar Typhi Quailes strain were seen, as shown in Figure 2. Three independent experiments found that the *fliC-d *gene of *Salmonella *serovar Typhi in the DNA preparations made from the cultures after 3 to 5 hours of incubation was detected by PCR, but not in those made from the cultures incubated for less than 3 hours (see Table 2). Taking account of the fact that 10 μl of the 100 μl DNA preparations made from the cultures was used in each PCR reaction, and the *fliC-d *amplicons were only seen in the cultures with more than 105 CFU of *Salmonella *serovar Typhi shown in Table 2, the limit of detection for this assay was about 10 CFU per PCR reaction. This study used a 4 ml blood sample spiked with 3 CFU of *Salmonella *serovar Typhi Quailes strain for the culture, and therefore the sensitivity of this blood culture-PCR method was equivalent to 0.75 CFU per millilitre of blood. At least three hours of incubation were needed for the bacteria to be enriched to a detectable level with a regular PCR.

**Table 2:** The growth and PCR detection of *Salmonella *serovar Typhi in TSB-bile blood culture *.

Incubation time (hour)	CFU^a^	*filC-d *amplicons^b^
0	3	- - -

1	4	- - -

2	17	- - -

3	105	+++

4	209	+++

5	4461	+++

*Three bacteria of *Salmonella *serovar Typhi were incubated in the TSB-bile blood culture containing 2.4% ox bile and 20% blood. ^a ^The mean of three independent experiments; ^b ^*Salmonella *serovar Typhi *fliC-d *amplicons resulting from PCR using the DNA templates prepared from three independent cultures.

## Discussion

Blood culture is currently the gold standard for diagnosis of typhoid fever, but it takes several days for the isolation and identification of causative organisms. As early diagnosis of the disease and prompt treatment are essential for optimal management, especially in children, a rapid sensitive detection method for typhoid fever is urgently needed. PCR is sensitive and rapid, and could possibly replace blood culture as the new "gold standard". However, initial research indicated similar sensitivity to blood culture and lower specificity [23].

Given the problems associated with conventional methods for diagnosis of typhoid fever, the present study was carried out in an attempt to develop a rapid, reliable, specific, and sensitive method for detection of *Salmonella *serovar Typhi. Artificially spiked blood samples were used throughout the study to mimic clinical specimens in the development and evaluation of a blood culture PCR method for detection of *Salmonella *serovar Typhi.

Early studies found that the number of *Salmonella *bacteria circulating in the blood of patients with *Salmonella *bacteremia is small, e.g. 0.5-22 bacteria per millilitre of blood found in 15 patients with typhoid fever [19], fewer than 2 bacteria per millilitre of blood found in 90% of 80 positive blood cultures from patients with *Salmonella *bacteremia [11], and a median of 0.3 (IQR, 0.1-10) bacteria per millilitre of blood in further 81 patients with typhoid fever [8]. Therefore, it is very important to use small bacterial inocula when developing and evaluating a system for detection of *Salmonella *serovar Typhi in clinical specimens. In the present study, blood samples were spiked with *Salmonella *serovar Typhi Quailes strain at a ratio of 1 ± 0.5 CFU per millilitre of blood, which closely mimics clinical specimens taken from patients with typhoid fever.

The initial evaluation study, in which 2-6 CFU of *Salmonella *serovar Typhi Quailes strain were mixed with 4 ml of blood and then cultured in TSB medium, found that the bacteria did not grow during 5 hours of incubation. The results remained the same when blood samples taken from different healthy donors were used. Moreover, when blood was artificially inoculated with a small number of *Salmonella *bacteria, followed by plating, fewer bacteria were recovered from the inoculated blood samples than the input (data not shown). This, consistent with other studies [11,19,24,25], clearly demonstrates that human blood has bactericidal activity against *Salmonella *serovar Typhi. Furthermore, one study found that the defibrinated whole blood from patients with *Salmonella *bacteremia was bactericidal for small inocula of three of five *Salmonella *strains isolated from the patient's blood [24]. This suggests that the blood or serum of patients with *Salmonella *bacteremia is also frequently bactericidal against the *Salmonella *isolated from their blood. Therefore, a culture medium which inhibits the bactericidal activity of blood may be beneficial in the development of a blood culture-PCR assay system.

Since bile was first used for blood culture of patients with *Salmonella *bacteremia in 1906 [26], bile containing media have repeatedly been found superior for isolation of enteric fever pathogens (*Salmonella *serovars, Typhi and Paratyphi) from whole blood [11]. Conradi attributed the effectiveness of bile as a culture medium to its effect in preventing coagulation of blood [26], and further demonstrated that bile decreases the bactericidal activity of serum [27]. Although Kaye et al. did not find that the anticoagulant effect was an important factor in the superiority of bile over TSB as a blood culture medium for *Salmonella*, they confirmed and expanded the observation that bile decreases the bactericidal activity of serum [11]. They found that bile inactivated the complement activity of blood, and also disrupted leukocytes in which *Salmonella *may be present. They argued that in patients with *Salmonella *bacteremia rapid release of *Salmonella *from leukocytes by bile might be a very important factor contributing to the advantage of bile as a culture medium. The intracellular presence of *Salmonella *serovar Typhi was demonstrated by the study [8] showing that the number of CFU of *Salmonella *serovar Typhi per milillitre sample increased by 3.3 fold and 1.9 fold respectively when bone marrow and blood cells were lyzed. Therefore, a blood culture medium could increase bacterial growth if it contains blood cell lyzing agents. Having examined further the effect of bile on the growth of *Salmonella *serovar Typhi, the present study confirmed the observation that bile does not have a growth-promoting effect on *Salmonella *[11]. In fact, *Salmonella *serovar Typhi Quailes strain did not survive in 10% bile in the absence of TSB (data not shown). Furthermore, the present study also demonstrated that bile, in absence of blood, inhibits the growth of *Salmonella *serovar Typhi Quailes strain at high concentrations of bile in TSB-bile medium. In order to determine the concentration of bile for use in a TSB-bile blood culture system, the lysis of blood cells by bile was studied. The results showed that the lysis of blood cells depends on the bile concentration and incubation time. At a concentration of 2.4%, bile not only lyzes blood cells completely within 1.5 hours so that the intracellular bacteria could be released, but also has no inhibiting effect on the growth of *Salmonella *serovar Typhi Quailes strain. Therefore, 2.4% bile seems an optimal concentration for use in the TSB-bile blood culture system.

It has been asserted that PCR is sensitive and rapid, and is a better alternative than conventional methods for pathogen detection. In the last decade, PCR has been widely researched for early diagnosis of typhoid fever. PCR as a diagnostic tool for detection of *Salmonella *serovar Typhi was first studied by Song et al. [12] who developed a nested PCR for amplification of the *fliC-d *gene of *Salmonella *serovar Typhi which could detect 5 bacteria/ml, compared to10^6 ^bacteria/ml by regular PCR. Recently, Ali et al. [18] reported a nested multiplex PCR for detection of both *Salmonella *serovars, Typhi and Paratyphi, with a sensitivity of 10 bacteria/ml. However, initial research indicated that PCR has similar sensitivity to blood culture and lower specificity [23]. This, at least in part, can be attributed to the low number of *Salmonella *bacteria circulating in the blood of typhoid patients and the inadequate method of DNA preparation. In normal clinical samples, there is a median of 0.3 CFU per millilitre of blood which is far below the sensitivity of PCR so far developed, and therefore, it is not surprising that PCR has not been used for diagnosis of typhoid fever. To overcome the difficulties caused by the low numbers of *Salmonella *bacteria present in typhoid patient blood samples, pre-enrichment of bacteria is necessary prior to PCR detection. A recent study demonstrated that the 5 hour broth culture enrichment improved PCR sensitivity by 10 times for spiked blood, and 100 times for spiked stool samples [28]. The present study has used the optimized TSB-bile blood culture system, in which 0.75 bacteria per ml of blood was used to mimic real clinical samples, and 4 ml blood for the culture with 3 CFU in total, for enrichment of bacteria prior to the PCR detection of *Salmonella *serovar Typhi. At the bacterial level used in present study, it was very difficult to detect the presence of *Salmonella *serovar Typhi by regular PCR without bacterial enrichment and/or removal of human DNA. However, three hour enrichment of *Salmonella *serovar Typhi in the TSB-bile medium could increase the number of CFUs to the level at which detection is possible using regular PCR. The sensitivity of this novel blood culture PCR method was equivalent to 0.75 CFU per millilitre of blood which is comparable to the bacterial number present in clinical typhoid samples. Moreover, the turnaround time of the assay was less than 8 hours rather than several days for conventional blood culture. Therefore, this new TSB-bile blood culture PCR system is superior (speed and sensitivity) to conventional blood culture and PCR methods and could potentially make early detection of *Salmonella *serovar Typhi possible for prompt treatment of patients with typhoid fever.

## Competing interests

AJP acts as chief and principal investigator for clinical trials conducted on behalf of Oxford University, sponsored by vaccine manufacturers (Novartis Vaccines, GlaxoSmithKline, Sanofi-Pasteur, Sanofi-Pasteur MSD, and Wyeth Vaccines), but does not receive any personal payment from them. Industry sourced honoraria for consultancy, lecturing or writing and travel expenses and grants for educational activities are paid directly to an educational/administrative fund held by the Department of Paediatrics, University of Oxford or an independent charity.

## Authors' contributions

LZ and AJP conceived of the study and carried out its design. LZ performed the assays and drafted the manuscript. AJP edited the manuscript. All authors read and approved the final manuscript.
